# Chondroid Syringoma of the Thenar Eminence in a US Veterans Administration (VA) Patient

**Published:** 2021-05-11

**Authors:** Muntazim Mukit, Ibrahim Ortanca, Nina Krassilnik, Kalyan Dadireddy

**Affiliations:** Memphis Veterans Administration Medical Center (VAMC), Memphis, Tenn; and University of Tennessee Health Science Center (UTHSC), Memphis

**Keywords:** thenar eminence, Veterans Administration, chondroid syringoma, hand mass, hyaline cell-rich chondroid syringoma variant

## DESCRIPTION

A 56-year-old right-hand-dominant man with a history of hypertension presented with a left thenar eminence cystic mass that started 2 months earlier. The patient denied symptoms, trauma, drainage, or pain but was concerned as he had had a tumor removed from his left forearm previously. The mass was stable in size. On examination, the mass was 1.0 × 0.5 cm. The digits on the patient's left hand were neurovascularly intact; the patient had full range of motion and normal hand cascade. The differential included schwannoma versus ganglion cyst. The patient underwent operative excision and had an uneventful postoperative course. Pathology showed “acral skin with an encapsulated nodular tumor in the dermis, composed of a few ducts, some adipocytes and sheets of ‘plasmacytoid’ cytoplasm, focally residing in a myxoid stroma…. The appearances are consistent with a hyaline (myoepithelial) cell-rich mixed tumor or skin.” The case report was deemed institutional board review exempt.

## QUESTIONS

1. What is chondroid syringoma?
2. How does chondroid syringoma present?
3. What are its histological features?
4. What are the treatment and prognosis?

## DISCUSSION

Chondroid syringomas are mixed tumors of the skin composed of epithelial and mesenchymal elements.^1^ Hirsch and Helwig first introduced the term in 1961 since these lesions have both epithelial and stromal elements.^2^ Their criteria for diagnosis were as follows: (1) nests of cuboidal or polygonal cells; (2) intercommunicating tubuloalveolar structures lined with 2 or more rows of cuboidal cells; (3) ductal structures composed of 1 or 2 rows of cuboidal cells; (4) occasional keratinous cysts; and a (5) matrix of varying composition.^2^^-^4 A variant of these lesions has been called hyaline cell-rich chondroid syringoma (HCRCS).^5^ The incidence is said to be 0.01% to 0.098%.^4^

Chondroid syringoma usually presents in the head and neck region.^1^^,^^3^ The mass is slow-growing, firm, and nontender and may present as a papule, subcutaneous nodule, or a cyst ranging in size from 0.5 to 3.0 cm.^1^^,^^3^ The lesions are typically adherent to the skin and usually occur in adults older than 35 years.^6^ The male to female ratio is reported to range from 2:1 to 5:1.^1^^,^^3^^,^^6^ In the literature, chondroid syringomas have been reported in the hand but its occurrence is still rare. One report states that there have been only 22 patients who have chondroid syringoma in the hand.^1^ Another report found that the HCRCS variant has a predilection for the extremities, consistent with our patient's presentation.^5^ Clinically, these lesions may be confused with sebaceous cysts, dermoid cysts, or neurofibromas.^7^

On histology, these tumors appear similar to benign salivary gland mixed tumors.^1^ The cells have eosinophilic hyaline stroma and plasmacytoid features.^5^ The stroma may be myxoid, chondroid, fibrous, adipocytic, or osseous.^1^ Headington^8^ categorized chondroid syringomas into 2 types, apocrine and eccrine. The eccrine type consists of small, round tubules that are evenly spaced in a myxoid-chondroid matrix, while the apocrine type consists of irregularly branching tubules lined by thicker epitheliaum.^1^ The HCRCS variant consists of plasmacytoid cells with hyaline cytoplasm in a myxochondroid to hyaline stroma and stains positive for cytokeratin, vimentin, and S100, with inconsistent expression of myoepithelial differentiation markers such as actin and calponin.^5^ Our patient's mass had a similar histological appearance and immunohistochemical profile, staining positively for cytokeratin and S100 and focally for calponin.

The treatment of chondroid syringoma is excision. Excision is also diagnostic.^3^ Recurrence happens secondary to incomplete excision.^1^^,^^6^ Including some normal tissue with excision can help guarantee complete removal.^4^ Most lesions are benign. Malignant chondroid syringomas are thought to arise de novo and not from existing benign lesions.^3^ Malignant lesions are usually larger, more commonly seen in women, and located on the extremities or the trunk, with a 1:2 male to female ratio and average age of diagnosis of 48 years.^1^^,^^3^^,^^6^ Malignant lesions may invade locally and/or metastasize to lymph nodes, bone, or lung.^6^ Some reports state that cytologic atypia, tumor necrosis, several mitosis, poorly differentiated chondroid elements, and an excessive mucoid matrix are suggestive of malignancy, but other reports have found that lesions may not have any of these features and still be malignant.^3^^,^^6^

## SUMMARY

Chondroid syringoma is a benign mixed tumor of the skin.^1^ The HCRCS variant consists of plasmacytoid cells in a myxochondroid/hyaline stroma and stains for cytokeratin, S100, vimentin, and sometimes actin and calponin.^5^ The HCRCS variant appears on extremities. Surgical excision is diagnostic and curative. Recurrence occurs due to incomplete excision.^1^^,^^6^

## Figures and Tables

**Figure 1:**
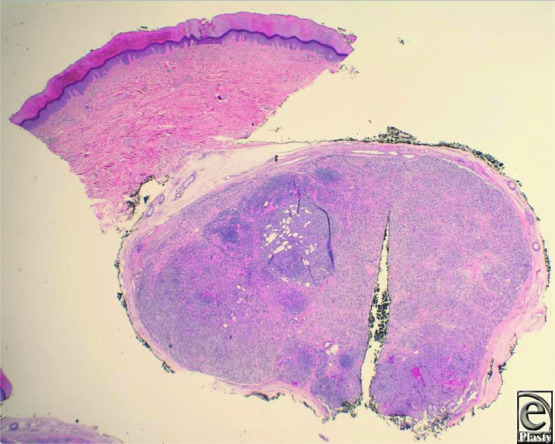
Low-power image showing a well-circumscribed, encapsulated tumor in subcutis (H&E, original magnification × 2).

**Figure 2:**
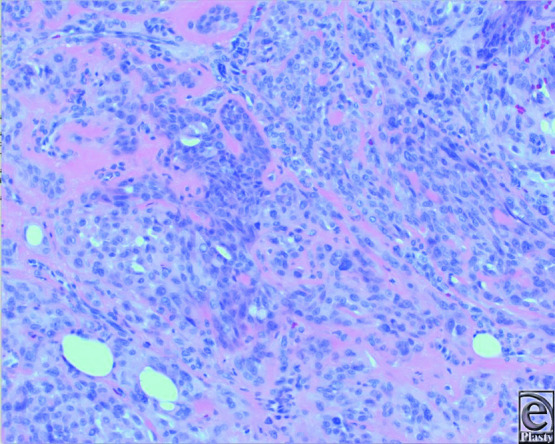
Tumor is composed of epithelioid cells with abundant eosinophilic cytoplasm, representing myoepithelial cells. Scattered are a few ducts lined by cuboidal epithelial cells. The stroma is hyalinized (H&E, original magnification × 10).

**Figure 3:**
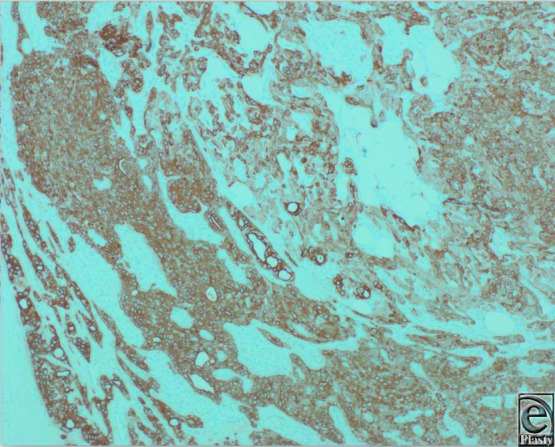
Broad-spectrum cytokeratin stains both epithelial and myoepithelial cells. Epithelial cells and staining ducts are more crisp (IHC for AE1/3, original magnification × 10).

**Figure 4:**
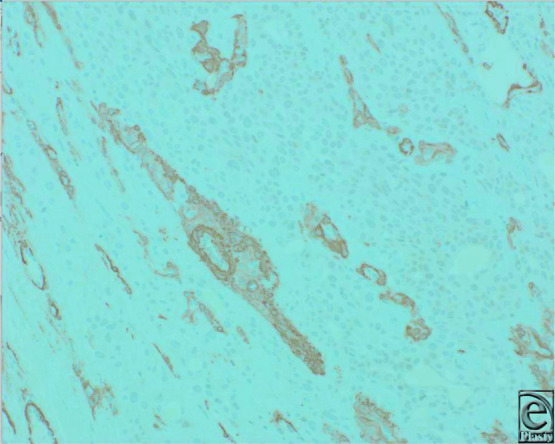
SMA immunohistostaining highlights the myoepithelial cells around ducts.

**Figure 5:**
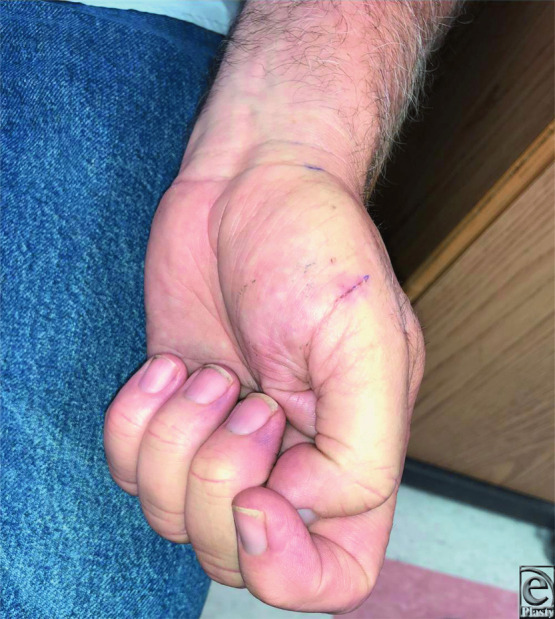
Postoperative day 9 anteroposterior photograph of the left hand showing a well-healed incision of thenar eminence.
